# The Invention of Spectacles

**Published:** 1899-10

**Authors:** 


THE *British Medical Journal* says that the invention of spectacles is often attributed to Roger Bacon, who died in 1294. Further research, however, has shown that in 1285, Savrino Degli Armati, a Florentine, worked glass into the form of a lens as a help to vision. For him, therefore, may justly be claimed the honor of having invented spectacles. He died in Florence in 1317, and was buried in the Church of Santa Maria Maggiore.

